# Impact of political partisanship on public interest in infection prevention measures in the United States: An infodemiological study

**DOI:** 10.1016/j.pmedr.2021.101493

**Published:** 2021-07-14

**Authors:** Christopher Y.K. Williams, Alice F. Ferreira

**Affiliations:** University of Cambridge, School of Clinical Medicine, Addenbrooke's Hospital, Hills Rd, Cambridge CB2 0SP, United Kingdom

**Keywords:** Public health, Infection prevention, Political partisanship, Covid-19, Infodemiology, Google Trends

## Abstract

There has been conflicting public messaging from government and state officials about recommended health behaviours during the COVID-19 pandemic. We examined whether differences in political affiliation influences the public’s interest in infection prevention measures in the United States. State-specific data on public search interest in four key infection prevention measures (Quarantine, Social distancing, Hand washing and Masks) were obtained from Google Trends for the period 1 January 2020 to 12 December 2020. Political affiliation was ascertained based on the 2020 U.S. Presidential election results and 2017 Cook Partisan Voting Index. Spearman’s rank, partial correlation, and multiple regression analyses were conducted to compare political partisanship with public interest in infection prevention measures and overall case rate per 100 000 population. Statistical analysis was performed in R version 4.0.3.

The COVID-19 pandemic has led to significantly increased public interest in infection prevention measures. The greater the support for the Democratic Party, the greater the search interest in all four measures analysed. Political partisanship was most highly correlated with searches relating to Quarantine (ρ = 0.79, *p* < 0.001), followed by Social distancing (ρ = 0.71, *p* < 0.001), Hand washing (ρ = 0.69, *p* < 0.001), and Masks (ρ = 0.66, *p* < 0.001). These findings were robust to using two different partisanship measures, controlling for state-level demographic variables, different pandemic onset dates, and using *exact* rather than *Topic* search methods. This partisan divide among the American people has important health implications that must be better addressed. We call for clear, bipartisan support of simple public health advice to combat the continued SARS-CoV-2 spread across the USA.

## 1 Introduction

The COVID-19 pandemic has seen significant controversy regarding compliance with public health measures in the United States. Adherence to social distancing and wearing of face masks have become politically charged issues, despite their recommendation in the Centers for Disease Control and Prevention’s (CDC) guidelines (CDC. Coronavirus Disease, 2019). An increasing body of evidence suggests that the public’s response to the pandemic is strongly influenced by their political viewpoint. Early surveys investigating attitudes towards COVID-19 found that Democrats had more knowledge and concern about COVID-19 (Clements, 2020, Clinton et al., 2021), reduced likelihood of attending large gatherings (Clements, 2020), and held greater trust in the efficacy of social distancing than their Republican counterparts (Allcott et al., 2020). Similarly, analyses of mobile phone data have revealed that areas with more Republican voters were associated with significantly less social distancing (Allcott et al., 2020, Hsiehchen et al., 2020, Gollwitzer et al., 2020).

Whilst the relationship between partisanship and social distancing has been closely studied (Clinton et al., 2021, Allcott et al., 2020, Hsiehchen et al., 2020, Gollwitzer et al., 2020), less is known about public interest in the other key CDC recommendations, principally hand washing, self-quarantine and wearing a face mask. We analysed data from Google Trends to investigate the effect of political affiliation on public search interest in all four of these COVID-19 infection prevention measures. Infodemiological methods are increasingly used to explore public behaviour, providing real-time information on search trends and the evolution of public interest and awareness over time (Bernardo et al., 2013, Walker et al., 2020, Brodeur et al., 2021). We hypothesised that certain political affiliations would be associated with decreased public search interest in the four main infection prevention measures.

## 2 Methods

Using Google Trends (https://trends.google.com/trends), we examined the relative search interest for four key COVID-19 infection prevention measures: Quarantine, Social distancing, Hand washing and Masks. These represent the main COVID-19 infection prevention measures advised by the CDC (CDC. Coronavirus Disease, 2019). We searched these items as *Topics*, which encompasses all terms that share the same concept regardless of language. Google Trends was used as it represents one of the most prominent Web-based surveillance tools that can capture the concerns and interests of the general public. Full search documentation can be found in Supplementary File 1. The checklist provided by Nuti et al. (2014) was used as a basis for search strategy reporting (Table S1).

### 2.1 Measures

State-specific data were retrieved from the “Interest by sub-region” section of Google Trends for the period 1 January 2020 to present day (12 December 2020) and downloaded in comma-separated values format. 1 January 2020 was selected as the start of the search period since the Huanan Seafood Market in Wuhan, China was closed for disinfection from this date (WHO | Novel Coronavirus – China. WHO. Accessed December 20, 2020). Additional analysis of the period 20 January 2020 to present day, corresponding to the first recorded case of SARS-CoV-2 in the United States, was also conducted (Figure S1, Table S2). Search term popularity in each state is relative to the total number of Google searches performed over a specified time and is reported as “Relative Search Volume” (RSV). Data from each state are further standardised to the state with the highest relative search popularity. This allows the ranking of relative search popularity in a manner proportional to the state with the highest popularity, which is given an RSV of 100. We additionally assessed the four main keywords as exact searches, with similar findings (Tables S3, S4; Figure S2).

In order to examine differences in search interest between states as the pandemic progressed, we divided the pandemic into three distinct, pre-specified phases. We used the World Health Organisation (WHO) Dashboard of confirmed COVID-19 cases to identify the peaks and troughs of weekly U.S. COVID-19 cases (United States of America: WHO Coronavirus Disease (COVID-19) Dashboard With Vaccination Data, 2021). 25 May 2020 and 7 September 2020 were confirmed as the two principal troughs separating the first–second, and second-third, waves of U.S. COVID-19 cases respectively. Google Trends data was extracted in a similar manner to above for the periods 1 January 2020 to 25 May 2020 (1st wave), 25 May 2020 to 7 September 2020 (2nd wave), and 7 September 2020 to 12 December 2020 (3rd wave to present day).

The 2017 Cook Partisan Voting Index (PVI) (Introducing the, 2017) was used as a measure of how strongly a state affiliates with the Democratic or Republican Party, compared to the nation as a whole. It is calculated based on how each state voted in the previous two Presidential elections (2012 and 2016). A PVI score of D + 4, for example, means that in the 2012 and 2016 elections, that state performed an average of four points more Democratic than the nation did as a whole. We standardised this score for the purpose of correlation analysis, such that positive values represent Democratic-leaning states and negative values represent Republican-leaning states. State voting results in the 2020 U.S. Presidential election were used to provide a more up-to-date representation of a state's overall political lean. Data on cumulative COVID-19 cases recorded in each state were retrieved from the Centers for Disease Control and Prevention up to 12 December 2020 (CDC. COVID-19 Cases, Deaths, and Trends in the US | CDC COVID Data Tracker. Centers for Disease Control and Prevention, 2020).

The following state-level demographic attributes were obtained from the 2018 CDC Behavioural Risk Factor Surveillance System (BRFSS) for use as pre-specified control variables: gender (% males), household income (proportion earning >$50000), age (proportion > 45 years) and race (% White, non-hispanics) (Prevalence Trends, 2019). Data on state-specific internet use (proportion who have used the internet in the past 30 days) was obtained from the 2017 BRFSS as this is the most currently available data (Prevalence Trends, 2019). State population density was used as an additional control, calculated by dividing state population estimates (2019 Census data) (State, 2021) by land area in square miles (Bureau, 2021).

### 2.2 Statistical analysis

Spearman’s rank was used to determine correlation coefficients (Spearman’s ρ) and *p* values. Secondary analyses were performed using partial correlation and multivariable linear regression to control for demographic third variables. Independent t-tests were used to compare continuous variables for RSV among states which voted for Biden vs Trump. Mean RSV for Biden- vs Trump-supporting states were calculated from the same data used in correlation analyses. The RSV of each Biden- or Trump-supporting U.S. state grouped by 2020 Presidential election outcome was averaged and reported for each search term as *Mean RSV*. Statistical analysis was performed using R version 4.0.3. *p* value < 0.05 was significant.

## 3 Results

**Fig. 1:**
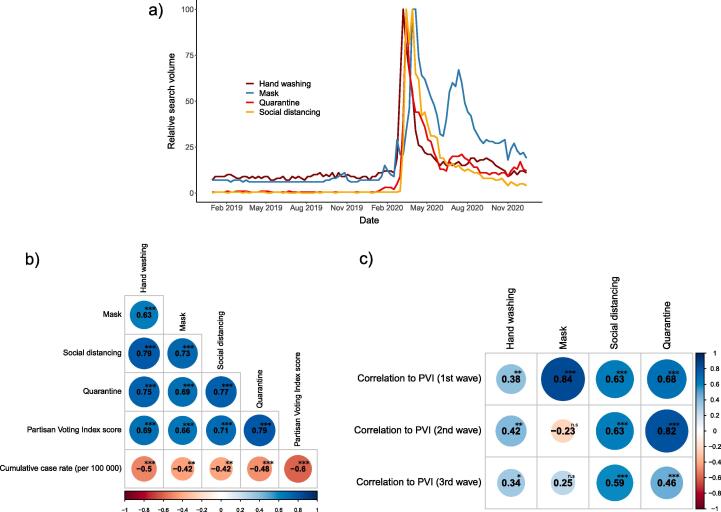
a) Time series of Relative Search Volume (RSV) for key public health measures (*Social distancing, Hand washing, Masks, Quarantine*) between 1 January 2019 and 12 December 2020. b) Spearman’s rank correlation matrix comparing Relative Search Volume (RSV) of key public health measures (*Hand washing, Masks, Social distancing, Quarantine*) to the 2017 Cook Partisan Voting Index (PVI) and cumulative COVID-19 case rate (per 100 000 population). Correlation coefficients (ρ) represented graphically and numerically. ^a^*p* = 0.002; *p* < 0.001 for all other correlation coefficients. c) Spearman’s rank correlation table comparing Relative Search Volume (RSV) and 2017 Cook Partisan Voting Index (PVI) for the three distinct *“waves”* of COVID-19 cases in the United States. Correlation coefficients (ρ) represented graphically and numerically. 1st wave = 1 January 2020 to 25 May 2020; 2nd wave = 25 May 2020 to 7 September 2020; 3rd wave = 7 September 2020 to 12 December 2020 (present day). * p < 0.05, ** p < 0.01, *** p < 0.001; n.s – not significant.

The COVID-19 pandemic has seen a significant increase in searches for terms relating to Quarantine, Social distancing, Hand Washing and Masks in the United States (Fig. 1a). As expected, states with greater relative search volumes for one of these four topics were significantly more likely to register higher search interest for the other topics relating to infection prevention (Fig. 1b).

Next, search interest and cumulative COVID-19 case number per 100 000 were analysed in relation to the 2017 Cook PVI across all states. We found that the more Democratic-leaning a state, the higher the relative search interest for all four infection prevention measures analysed (Fig. 1b). Political partisanship was most strongly correlated with searches relating to Quarantine (ρ = 0.79, *p* < 0.001), followed by Social distancing (ρ = 0.71, *p* < 0.001), Hand washing (ρ = 0.69, *p* < 0.001), and Masks (ρ = 0.66, *p* < 0.001). Greater search interest in infection prevention measures was associated with lower reported SARS-CoV-2 case rates (Fig. 1b). All correlations achieved statistical significance. These findings are supported by *t*-test analyses of mean Relative Search Volumes among states which voted for Biden vs Trump in the 2020 U.S. Presidential election. Biden-supporting states were found to have significantly greater search interest for the four infection prevention measures compared to their Trump-supporting counterparts (Table 1a).

**Table 1:** Differences in mean Relative Search Volume (RSV) of key public health measures between Trump- and Biden-supporting states in the 2020 U.S. Presidential Election. ^a^*p* values determined by independent *t*-tests. a) Whole analysis period = 1 January 2020 to 12 December 2020 (present day); b) 1st wave = 1 January 2020 to 25 May 2020; c) 2nd wave = 25 May 2020 to 7 September 2020; d) 3rd wave = 7 September 2020 to 12 December 2020.

	Period	Search term	Biden-supporting state (mean RSV [*SD*])	Trump-supporting state (mean RSV [*SD*])	*p* value^a^
a)	1 Jan 20 to 12 Dec 20 (whole period)	Social distancing	72.8 [10.9]	57.0 [8.4]	< 0.001
		Hand washing	69.5 [11.3]	57.7 [7.1]	< 0.001
		Mask	78.0 [5.9]	71.0 [3.1]	< 0.001
		Quarantine	53.5 [12.8]	38.7 [5.6]	< 0.001
b)	1 Jan 20 to 25 May 20 (1st wave)	Social distancing	74.5 [13.2]	57.0 [10.1]	<0.001
		Hand washing	73.7 [11.9]	66.9 [12.9]	0.055
		Mask	70.9 [8.9]	56.6 [4.6]	<0.001
		Quarantine	70.0 [10.0]	56.7 [8.1]	<0.001
c)	25 May 20 to 7 Sep 20 (2nd wave)	Social distancing	63.1 [12.1]	49.9 [11.0]	<0.001
		Hand washing	65.5 [10.7]	59.5 [12.9]	0.077
		Mask	79.1 [8.7]	81.6 [6.6]	0.247
		Quarantine	39.8 [17.6]	22.1 [6.1]	<0.001
d)	7 Sep 20 to 12 Dec 20 (3rd wave)	Social distancing	50.4 [7.8]	40.7 [15.6]	0.008
		Hand washing	43.7 [15.6]	39.0 [8.4]	0.181
		Mask	80.2 [8.0]	74.8 [9.5]	0.035
		Quarantine	44.5 [17.6]	32.1 [7.6]	0.002

Partial correlation analyses using 3rd variables controlling for state-specific demographics led to similar results (Table S5). Controlling for household income (proportion earning >$50000) led to the greatest decrease in strength of correlation (Social distancing ρ = 0.57, Hand washing ρ = 0.53, Mask ρ = 0.52, Quarantine ρ = 0.70; *p* < 0.001), while Spearman’s ρ was largely unchanged or increased when the remaining 3rd variables were controlled for. In addition, partisanship was more strongly associated with search interest in infection prevention measures than demographic variables in all comparisons but one (Table S6). In multivariable regression models controlling for the state-specific demographics above, the PVI score for each state remained significantly associated with RSV for each of the terms of interest (Tables S7-S10).

Lastly, we analysed partisan differences in searches for three distinct periods of the pandemic, corresponding to the three “waves” of COVID-19 cases across the United States (Fig. 1c*,* Table 1). We found a strong relationship between state partisanship and searches for Social distancing and Quarantine during the 1st and 2nd waves of the pandemic, with a weaker correlation (ρ = 0.59 and ρ = 0.46, respectively) for the 3rd wave. Independent *t*-test analyses confirmed significant partisan differences in search volume for Social distancing and Quarantine in all three sub-periods of the pandemic. In contrast, internet searches for Masks were strongly correlated with PVI in the 1st wave of the pandemic, but this relationship did not exist during subsequent waves. When search volume data for Hand washing was broken down into three phases, only moderate-weak correlations with state PVI were found, while differences in mean RSV between Biden- and Trump-supporting states did not reach statistical significance.

## 4 Discussion

This study identifies a significant association between political affiliation in the United States and public search interest in measures to prevent SARS-CoV-2 transmission. We found that the greater the support for the Democratic political party, the greater the search interest in four key COVID-19 infection prevention measures: Quarantine, Social distancing, Hand washing and Masks. Findings were robust to using two different partisanship measures, controlling for state-level demographic variables, different pandemic onset dates, and using *exact* rather than *Topic* search methods. When the data analysis period was broken down into three distinct phases, corresponding to the three *“waves”* of COVID-19 cases across the United States, the influence of political partisanship waned as time progressed. Notably, however, there remained strong-moderate correlations between partisanship and search interest in Social distancing and Quarantine even in the later stages of the pandemic.

These data support previous local survey and mobility studies on partisan differences in social distancing (Gollwitzer et al., 2020, Clements, 2020, Clinton et al., 2021, Allcott et al., 2020), and may reflect poor public health leadership among prominent government officials (Dying in a Leadership Vacuum., 2020). While the success of the vaccination roll-out may reduce the necessity for strict social distancing and mask-wearing (CDC, 2020), continued vigilance for COVID-19 symptoms and self-quarantine among individuals testing positive for the virus is imperative. Our findings emphasise the need for clear, bipartisan support of simple public health advice, particularly at the onset of a pandemic. Emerging evidence highlights the importance of state governor recommendations alongside national guidance - Democratic-learning counties responded more strongly to COVID-19-related recommendations from Republican, rather than Democratic, governors whose advice contradicted the national party line (Grossman et al., 2020). Future work should therefore consider how state and national health leaders might work together to better overcome these political divisions and improve public health for all.

## 5 Limitations

Our study has several limitations. Firstly, there is the lack of proven causal relationship between search interest and overall awareness of public health measures. Analysing Google Trends data relies on its representativeness of the general population and ability to accurately measure topic salience (Mellon, 2014). Internet users may not be representative of the general population, an issue we sought to mitigate by including internet use as a controlled variable. Validating the relationship between internet searches and public salience can be difficult. However, examination of Fig. 1a shows that the expected surge in infection prevention topic salience is appropriately captured by Google Trends, and others have used similar methods to capture other health-related topics (Walker et al., 2020, Brodeur et al., 2021, Barrios and Hochberg, 2020). Secondly, it is probable that public search interest in infection prevention measures is moderated by different variables with complex interactions, some of which may not have been adequately controlled for, despite our best efforts. Thirdly, greater awareness of health recommendations does not necessarily lead to increased compliance; observational studies of public behaviour are additionally required to build on our findings (Allcott et al., 2020, Gollwitzer et al., 2020). Lastly, this analysis was conducted at a state-level; hence, the heterogeneity of political ideology and overall search interest within specific districts/subregions may not be fully represented. Likewise, the nuances of individual-level political viewpoints have not been examined - people support political parties for different reasons and it is unlikely that our results hold true for all individuals with similar voting preferences.

## 6 Conclusions

To our knowledge, this is the first study to examine the relationship between political affiliation and public search interest in SARS-CoV-2 infection prevention measures across the United States. We found that the more Republican-leaning a state, the lower the search interest in recommended infection prevention measures. We call for greater tailoring of public health messages for people with different political views in order to better promote infection prevention behaviours and save lives.

## 7 Author statement

Christopher Williams: Conceptualisation, Data curation, Formal analysis, Investigation, Methodology, Supervision, Writing – original draft, Writing – review & editing.

Alice Ferreira: Formal analysis, Investigation, Validation, Visualisation, Writing – review & editing.

## Ethical approval

Ethical approval was not required as this study solely involved information that is freely available in the public domain.

## 9 Financial disclosure

This research did not receive any specific grant from funding agencies in the public, commercial, or not-for-profit sectors.

## 10 Data availability

All data is publicly available through Google Trends, the Behavioral Risk Factor Surveillance System (BRFSS), and the Centres for Disease Control and Prevention (CDC). Supporting R code is documented in Supplementary File 2.

## Declaration of Competing Interest

The authors declare that they have no known competing financial interests or personal relationships that could have appeared to influence the work reported in this paper.
