# Supplementary material for: Impact of political partisanship on public interest in infection prevention measures in the United States: An infodemiological study
Source: Prev Med Rep. 2021 Jul 14;23:101493. doi: 10.1016/j.pmedr.2021.101493 (PMC8326196; doi:10.1016/j.pmedr.2021.101493)
Supplement: Supplementary data 1 [file mmc1.pdf]

Supplementary File 1

Section/Topic	#	Checklist Item	Reported on Page #
**Search Variables**			
Access date	1	Provide the date(s) when Google Trends was accessed and when the data was downloaded.	Suppl. File 1
Time Period	2	Identify all the time periods that were searched for in Google Trends, providing up to the Month and Day in detail.	5
Query Category	3	Identify which query category was used for search; if not using a query category, designate that “all query categories were used”, which is the default setting.	Suppl. File 1
**Search Input**			
Full search Input	4	Provide the full search input(s) that were queried for in Google Trends, along with the appropriate documentation of search syntax (detailed in 4a and 4b). Ensure that the provision of the search input is clear, using brackets or other delineators to separate the search input from the body text.	5; Suppl. File 1
Combination	4a	If more than one search term was used, document whether those terms were used in combination with a plus sign (+), or if terms were excluded with a minus sign (-). If terms were not used in combination, state so clearly.	Suppl. File 1
Quotation Marks	4b	If there was more than one word in any search term, document whether those words were queried with quotation marks or not.	Suppl. File 1
**Rationale for Search Strategy**			
For Search Input	5	Provide the reasoning behind the choice of search input.	Suppl. File 1
For Settings Chosen	6	Provide the reasoning for the settings/search variables chosen to specify the search.	Suppl. File 1

Table S1. *Checklist for the Documentation of Google Trends Use*

Main analysis search terms [United States, All categories, Web search]: Quarantine (Topic), Social distancing (Topic), Hand washing (Topic), Mask (Topic). Web search was used to capture the volume of Google searches for each topic. Search terms were used in isolation and not combined. No quotation marks were required as “Topic” rather than “search term” were searched. Data were accessed and downloaded on 13 December 2020.

Google Trends *Topic* search allows all searches related to the specified search term to be captured, regardless of language. “Quarantine”, “Social distancing”, “Hand washing” and “Mask” were the main search terms as these represent four key COVID-19 infection prevention measures advised by the World Health Organisation and Centers for Disease Control and Prevention.[1,2] We chose not to consider other search terms in our main analysis due to the subjective nature of selecting search terms, which may have biased the results if terms were self-selected. Data on Relative Search Volumes (RSV) for all 50 states and District of Columbia (n = 51) were retrieved.

Search term popularity reflects the number of searches for that term relative to the total number of Google searches performed at the specified time (e.g from 1 January 2020 to 12 December 2020), in a specific location.[4] Although independent verification of the accuracy of searches mapped to each location has not been performed, the Trends interface provides data granularity down to metropolitan area, while the use of state-specific Google Trends data has been described by others previously.[3] Normalising by total number of Google searches ensures that differences in the total searches for each region are controlled for. Relative search volume (RSV) values for each state are then calculated by standardising each state’s search term popularity to the state with the greatest popularity. Google

Trends presents this data on a scale from 0 to 100, where an RSV of 100 is assigned to the location with the greatest search term popularity (as a fraction of total searches in that location for the specified time period), while a value of 50 indicates a location which has half the search term popularity.

The search period 1 January 2020 to present day (12 December 2020) was selected as the Huanan Seafood Market in Wuhan, China was closed for environmental sanitation and disinfection from 1 January 2020.[5] Although the first recorded case of SARS-CoV-2 in the United States was nineteen days later on 20 January 2020,[6] we anticipated that the rise in search interest for infection prevention measures would have occurred prior to this, as the world became more aware of the emerging virus threat. We have repeated our main analyses using the time period 20 January 2020 to present day (12 December 2020), with similar findings:

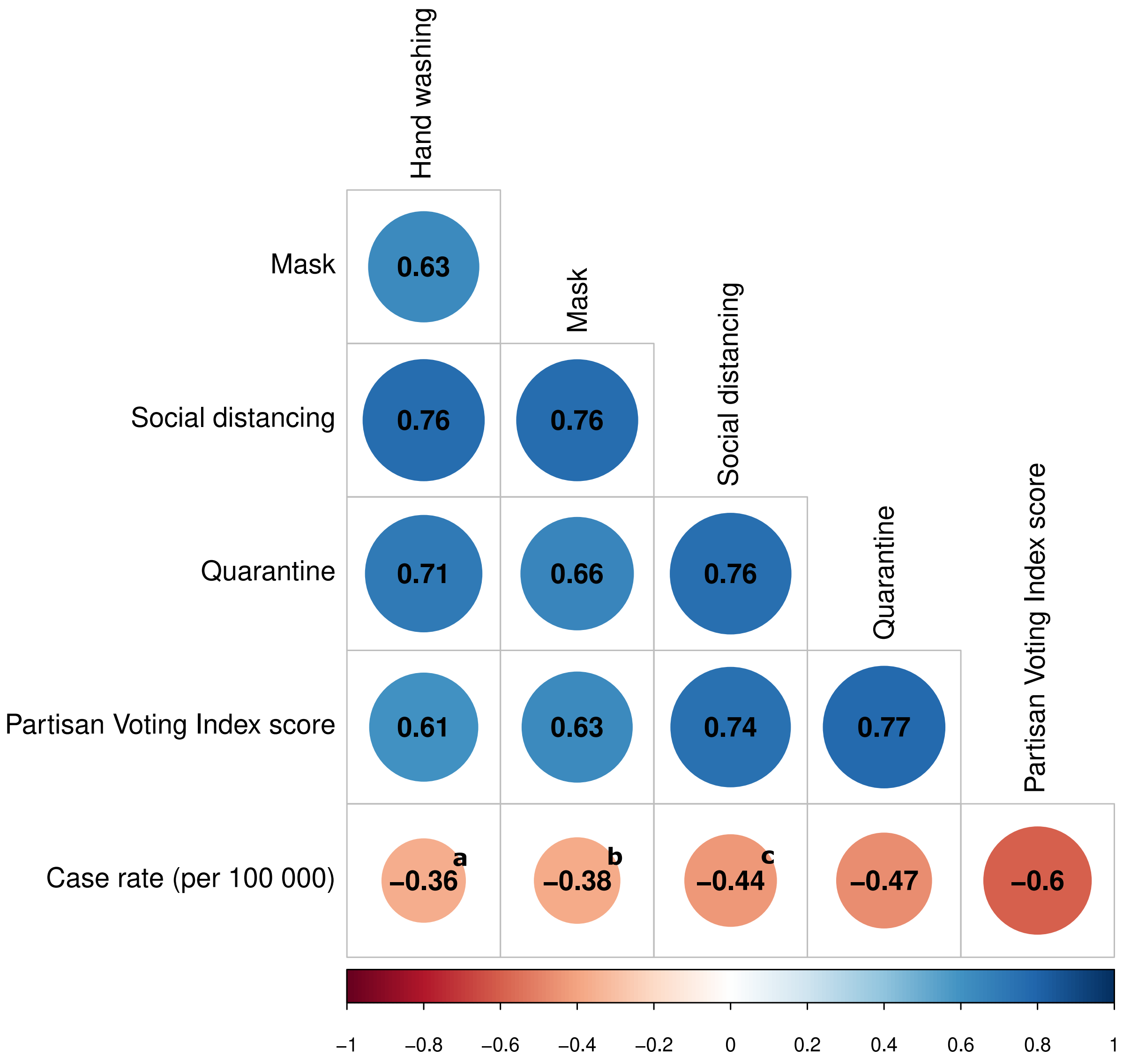

Figure S1. *20 January 2020 to 12 December 2020 analysis: Spearman's rank correlation matrix comparing Relative Search Volume (RSV) of key public health measures (Hand washing, Masks, Social distancing, Quarantine) to the 2017 Cook Partisan Voting Index (PVI) and cumulative COVID-19 case rate (per 100 000 population). Correlation coefficients (ρ) represented graphically and numerically.* [a]*p = 0.009;* [b]*p = 0.006;* [c]*p = 0.001; p < 0.001 for all other correlation coefficients.*

Search term	Biden-supporting state (mean RSV [*SD*])	Trump-supporting state (mean RSV [*SD*])	*p* value[a]
Social distancing	67.2 [12.0]	50.2 [7.8]	< 0.001
Hand washing	63.5 [11.4]	55.0 [11.5]	0.01
Mask	78.7 [6.0]	71.9 [4.1]	< 0.001
Quarantine	53.3 [12.9]	38.8 [6.3]	< 0.001

Table S2. *Differences in mean Relative Search Volume (RSV) of key public health measures (Topic search; period: 20 January 2020 to 12 December 2020) between Trump and Biden supporting states in the 2020 U.S Presidential Election. [a]p values determined by independent t tests*

***Topic* search vs *exact* search terms**

In order to better understand the nature of the Google Trends *Topic* search function, we extracted the Top 5 *Related queries* (defined by Google Trends as "*Users searching for your term also searched for these queries*") for each *Topic* search for the four main terms of interest (*"Quarantine" [Topic]*, *"Social distancing" [Topic]*, *"Hand washing" [Topic]* and *"Masks" [Topic]*) for the period 1 January 2020 to 12 December 2020 (*Table S3*). This helps confirm that the specified *Topic* searches relate to the main COVID-19 infection prevention terms in question.

We then sought to investigate whether similar findings would be obtained if the analyses presented in the manuscript were replicated using exact search terms (*"Quarantine" [search term]*, *"Social distancing" [search term]*, *"Hand washing" [search term]* and *"Masks" [search term]*), rather than *Topic* search. An advantage of using exact search terms, rather than *Topic* search, is that the method for aggregating related search terms into a *Topic* is not publicly defined. *Topic* search is therefore a less precise tool than the exact search function. However, this can also be an advantage since the inclusion of related search terms, which may be more frequently searched than the original search term, helps reduce the possibility of selection bias influencing the results (i.e *Topic* search better captures the *concept* rather than a particular keyword, which may not be the best keyword to use).

**Social distancing (Topic)**	**Hand washing (Topic)**
1. Distancing<br>2. Social distancing<br>3. Social distance<br>4. Coronavirus social distancing<br>5. Covid social distancing	1. Wash hands<br>2. Hand washing<br>3. Wash wash wash your hands<br>4. Wash your hands<br>5. Hand wash
**Mask (Topic)**	**Quarantine (Topic)**
1. Mask<br>2. Masks<br>3. Face mask<br>4. Face masks<br>5. N95	1. Quarantine<br>2. Covid quarantine<br>3. Coronavirus quarantine<br>4. Quarantine states<br>5. Quarantined

Table S3. *Top 5 Related queries for each Topic search for the four main terms of interest ("Quarantine" [Topic], "Social distancing" [Topic], "Hand washing [Topic] and "Masks" [Topic])*

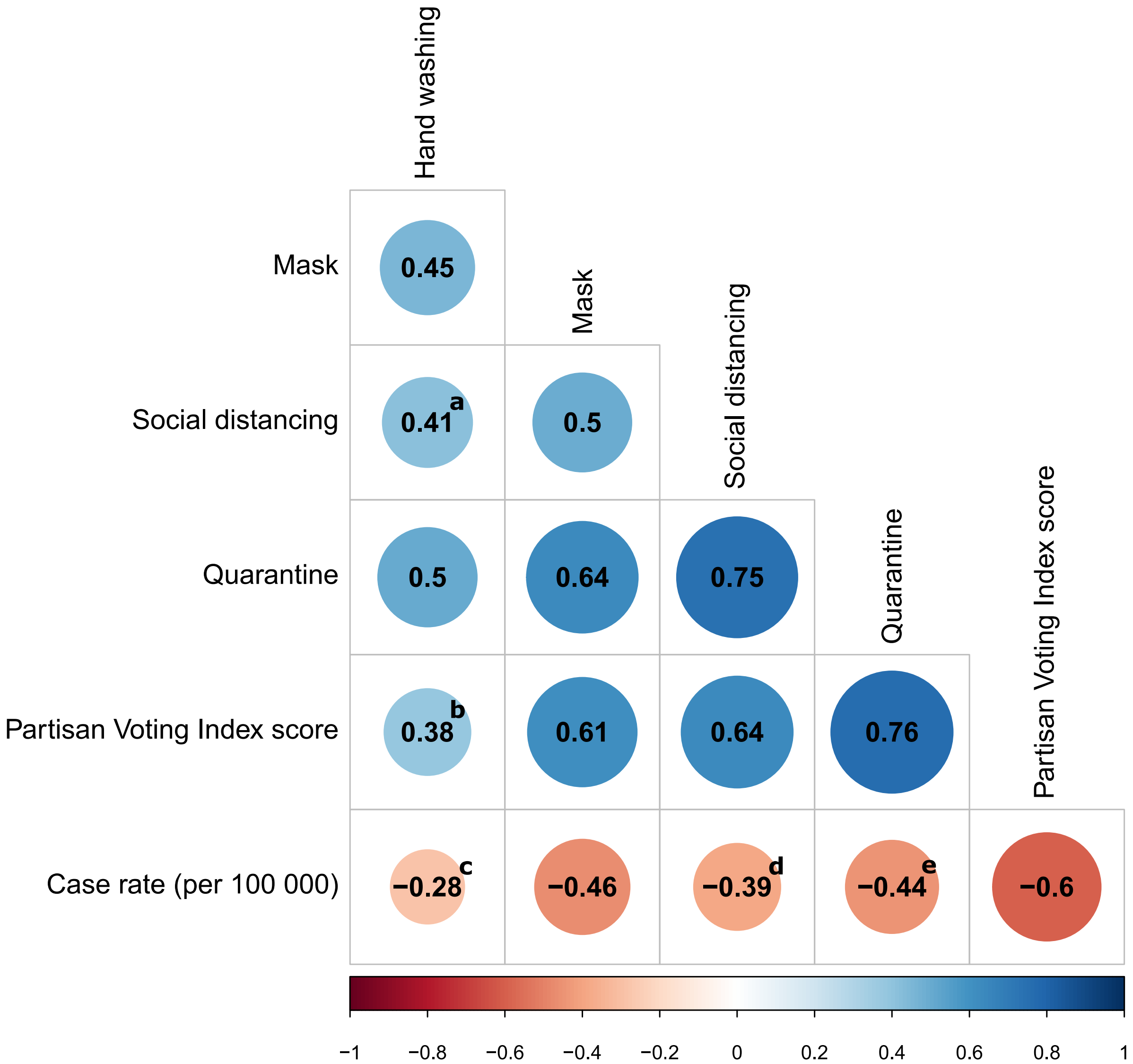

Figure S2. *Exact search term analysis: Spearman's rank correlation matrix comparing Relative Search Volume (RSV) of key public health measures (Hand washing, Masks, Social distancing, Quarantine) to the 2017 Cook Partisan Voting Index (PVI) and cumulative COVID-19 case rate (per 100 000 population). Correlation coefficients (ρ) represented graphically and numerically. [a]p = 0.003; [b]p = 0.005; [c]p = 0.046; [d]p = 0.005; [e]p = 0.001; p < 0.001 for all other correlation coefficients.*

**Search term**	**Biden-supporting state (mean RSV [*SD*])**	**Trump-supporting state (mean RSV [*SD*])**	***p* value[a]**
Social distancing	79.7 [11.9]	63.5 [8.5]	< 0.001
Hand washing	67.3 [15.4]	57.7 [12.0]	0.016
Mask	71.5 [6.4]	66.3 [2.6]	< 0.001
Quarantine	53.8 [13.0]	39.0 [5.9]	< 0.001

Table S4. *Differences in mean Relative Search Volume (RSV) of key public health measures (period: 1 January 2020 to 12 December 2020) between Trump and Biden supporting states in the 2020 U.S Presidential Election, using exact search terms. [a]p values determined by independent t tests.*

The above results suggest that a strong association remains between state partisanship and exact searches for *Quarantine*, *Social distancing* and *Masks* in particular. A weaker correlation between Partisan Voting Index score and searches for *Hand washing* was found when exact search term rather than *Topic* was searched. When mean RSV between Biden- and Trump-supporting states were compared using independent t tests, Biden-supporting states were again found to more frequently search for all four COVID-19 infection prevention measures.

Demographic control variables

Controlled 3rd variables	**Social distancing**	**Hand washing**	**Mask**	**Quarantine**
Internet use (% using the internet in past 30 days)	0.78***	0.69***	0.66***	0.80***
Race (% White, non-Hispanics)	0.85***	0.73***	0.81***	0.77***
Gender (% Male)	0.79***	0.74***	0.76***	0.82***
Age (% 45+ years)	0.70***	0.69***	0.65***	0.79***
Household income (% >$50000)	0.57***	0.53***	0.53***	0.70***
Population density (persons/sq mile)	0.71***	0.68***	0.68***	0.74***

Table S5. *Partial correlation analyses comparing Relative Search Volume (RSV) of key public health measures (Social distancing, Hand washing, Masks, Quarantine) to the 2017 Cook Partisan Voting Index, while controlling for the state demographics-related 3rd variables stated in the far-left column (Internet use, Race, Gender, Age, Household income, Population density). *** p < 0.001*

3rd variables	**Social distancing**	**Hand washing**	**Mask**	**Quarantine**
Internet use (% using the internet in past 30 days)	0.64***	0.49***	0.48***	0.41**
Race (% White, non-Hispanics)	0.13 n.s	-0.08 n.s	0.15 n.s	-0.29*
Gender (% Male)	0.09 n.s	0.03 n.s	0.14 n.s	-0.05 n.s
Age (% 45+ years)	0.22 n.s	0.06 n.s	0.42**	0.08 n.s
Household income (% >$50000)	0.78***	0.67***	0.55***	0.67***
Population density (persons/sq mile)	0.27 n.s	0.28 n.s	0.23 n.s	0.44**
Reference: Cook Partisan Voting index	0.71***	0.69***	0.66***	0.79***

Table S6. *Spearman's rank correlation analyses comparing Relative Search Volume (RSV) of key public health measures (Social distancing, Hand washing, Masks, Quarantine) to state demographics control variables (Internet use, Race, Gender, Age, Household income, Population density). * p < 0.05, ** p < 0.01, *** p < 0.001; n.s – not significant. Partisanship (Cook PVI) was more strongly associated with search interest in infection prevention measures than demographic variables in all comparisons but one (Social distancing vs Household income; ρ = 0.78).*

Univariable and multivariable regression models

Social distancing ~ Predictor variables	Univariable			Multivariable		
	Standardised coefficient (SE)	t value	*p* value	Standardised coefficient (SE)	t value	*p* value
PVI	0.74 (0.10)	7.49	< 0.001***	0.81 (0.12)	6.82	< 0.001***
Internet use	2.29 (0.42)	5.40	< 0.001***	0.47 (0.45)	1.03	0.307
Race	0.04 (0.11)	0.38	0.71	0.30 (0.07)	3.99	< 0.001***
Gender	-0.27 (1.79)	-0.15	0.88	2.50 (0.99)	2.53	0.015*
Age	-0.03 (0.54)	-0.06	0.95	0.30 (0.36)	0.84	0.404
Household income	1.30 (0.17)	7.46	< 0.001***	0.36 (0.19)	1.89	0.066
Population density	0.003 (0.00)	3.49	0.001**	0.001 (0.00)	1.44	0.158

Table S7. *Univariable and multivariable regression models comparing Relative Search Volume (RSV) for Social distancing (Topic) to the following predictor variables: Partisan Voting Index (PVI), Internet use (% using the internet in past 30 days), Race (% White, non-Hispanics), Gender (% Male), Age (% 45+ years), Household income (% >$50 000) and Population density (persons/sq mile). Multivariable model: Adjusted $R^2 = 0.86$; F-statistic = 45.29, p-value < 0.001. SE = standard error; * p < 0.05, ** p < 0.01, *** p < 0.001*

Hand washing ~ Predictor variables	Univariable			Multivariable		
	Standardised coefficient (SE)	t value	*p* value	Standardised coefficient (SE)	t value	*p* value
PVI	0.64 (0.09)	7.17	< 0.001***	0.82 (0.17)	4.89	< 0.001***
Internet use	1.29 (0.44)	2.95	0.005**	-0.62 (0.64)	-0.96	0.342
Race	-0.17 (0.10)	-1.76	0.084	0.09 (0.11)	0.89	0.376
Gender	0.72 (1.58)	0.45	0.653	5.19 (1.41)	3.68	< 0.001***
Age	-0.27 (0.47)	-0.58	0.567	0.05 (0.51)	0.10	0.919
Household income	0.90 (0.19)	4.85	< 0.001***	0.32 (0.27)	1.19	0.243
Population density	0.002 (0.00)	2.65	0.011*	-0.000 (0.001)	-0.07	0.942

Table S8. *Univariable and multivariable regression models comparing Relative Search Volume (RSV) for Hand washing (Topic) to the following predictor variables: Partisan Voting Index (PVI), Internet use (% using the internet in past 30 days), Race (% White, non-Hispanics), Gender (% Male), Age (% 45+ years), Household income (% >$50 000) and Population density (persons/sq mile). Multivariable model: Adjusted $R^2 = 0.64$; F-statistic = 13.77, p-value < 0.001. SE = standard error; * p < 0.05, ** p < 0.01, *** p < 0.001*

Mask ~ Predictor variables	Univariable			Multivariable		
	Standardised coefficient (SE)	t value	*p* value	Standardised coefficient (SE)	t value	*p* value
PVI	0.25 (0.06)	4.41	< 0.001***	0.29 (0.09)	3.06	0.004**
Internet use	0.69 (0.23)	3.01	0.004**	0.35 (0.36)	0.99	0.326
Race	-0.03 (0.05)	-0.50	0.623	-0.05 (0.06)	-0.85	0.401

Gender	0.55 (0.84)	0.66	0.513	1.90 (0.78)	2.42	0.020*
Age	0.56 (0.24)	2.34	0.023*	0.87 (0.28)	3.06	0.004**
Household income	0.40 (0.10)	3.85	< 0.001***	0.10 (0.15)	0.68	0.500
Population density	-0.000 (0.00)	-0.17	0.87	-0.000 (0.00)	-0.69	0.496

Table S9. *Univariable and multivariable regression models comparing Relative Search Volume (RSV) for Mask (Topic) to the following predictor variables: Partisan Voting Index (PVI), Internet use (% using the internet in past 30 days), Race (% White, non-Hispanics), Gender (% Male), Age (% 45+ years), Household income (% >$50 000) and Population density (persons/sq mile). Multivariable model: Adjusted R^2 = 0.60; F-statistic = 11.85, p-value < 0.001. SE = standard error; * p < 0.05, ** p < 0.01, *** p < 0.001*

**Quarantine ~ Predictor variables**	**Univariable**			**Multivariable**		
	**Standardised coefficient (SE)**	**t value**	***p* value**	**Standardised coefficient (SE)**	**t value**	***p* value**
PVI	0.72 (0.10)	7.11	< 0.001***	0.44 (0.19)	2.35	0.024*
Internet use	0.99 (0.51)	1.93	0.059	-0.67 (0.71)	-0.94	0.351
Race	-0.36 (0.10)	-3.59	< 0.001***	-0.28 (0.12)	-2.37	0.022*
Gender	-0.63 (1.77)	-0.36	0.724	3.77 (1.56)	2.42	0.020*
Age	-0.14 (0.53)	-0.26	0.796	1.11 (0.57)	1.97	0.056
Household income	0.99 (0.21)	4.77	< 0.001***	0.87 (0.30)	2.89	0.006**
Population density	0.002 (0.00)	2.20	0.032*	0.000 (0.00)	0.48	0.631

Table S10. *Univariable and multivariable regression models comparing Relative Search Volume (RSV) for Quarantine (Topic) to the following predictor variables: Partisan Voting Index (PVI), Internet use (% using the internet in past 30 days), Race (% White, non-Hispanics), Gender (% Male), Age (% 45+ years), Household income (% >$50 000) and Population density (persons/sq mile). Multivariable model: Adjusted R^2 = 0.65; F-statistic = 14.08, p-value < 0.001. SE = standard error; * p < 0.05, ** p < 0.01, *** p < 0.001*

Non-COVID-related hygiene/sanitary measures

We lastly sought to investigate the relationship between political partisanship and pre-specified non-COVID-related hygiene/sanitary measures. We hypothesised that non-COVID-related measures, which have not received the widespread public health coverage/messaging compared to COVID-related measures, would be less likely to be associated with state political partisanship.

The following search terms were used, with data extracted for the same search period as our main analyses (1 January 2020 to 12 December 2020): "Sanitary napkin" (Topic), "Oral hygiene" (Topic), "Teeth cleaning" (Topic), "Food safety" (Discipline). Data for "Antibiotics (Drug type) and "Antifungal" (Drug class) were also extracted – while not specifically a hygiene/sanitary measure, they provide additional insight into public interest in non-viral treatments. Similarly, "Health" (Topic) was included as a further control to evaluate each state's interest in overall health-related topics. The below results show that, as anticipated, searches for health topics unrelated to COVID-19 were only weakly correlated with political partisanship.

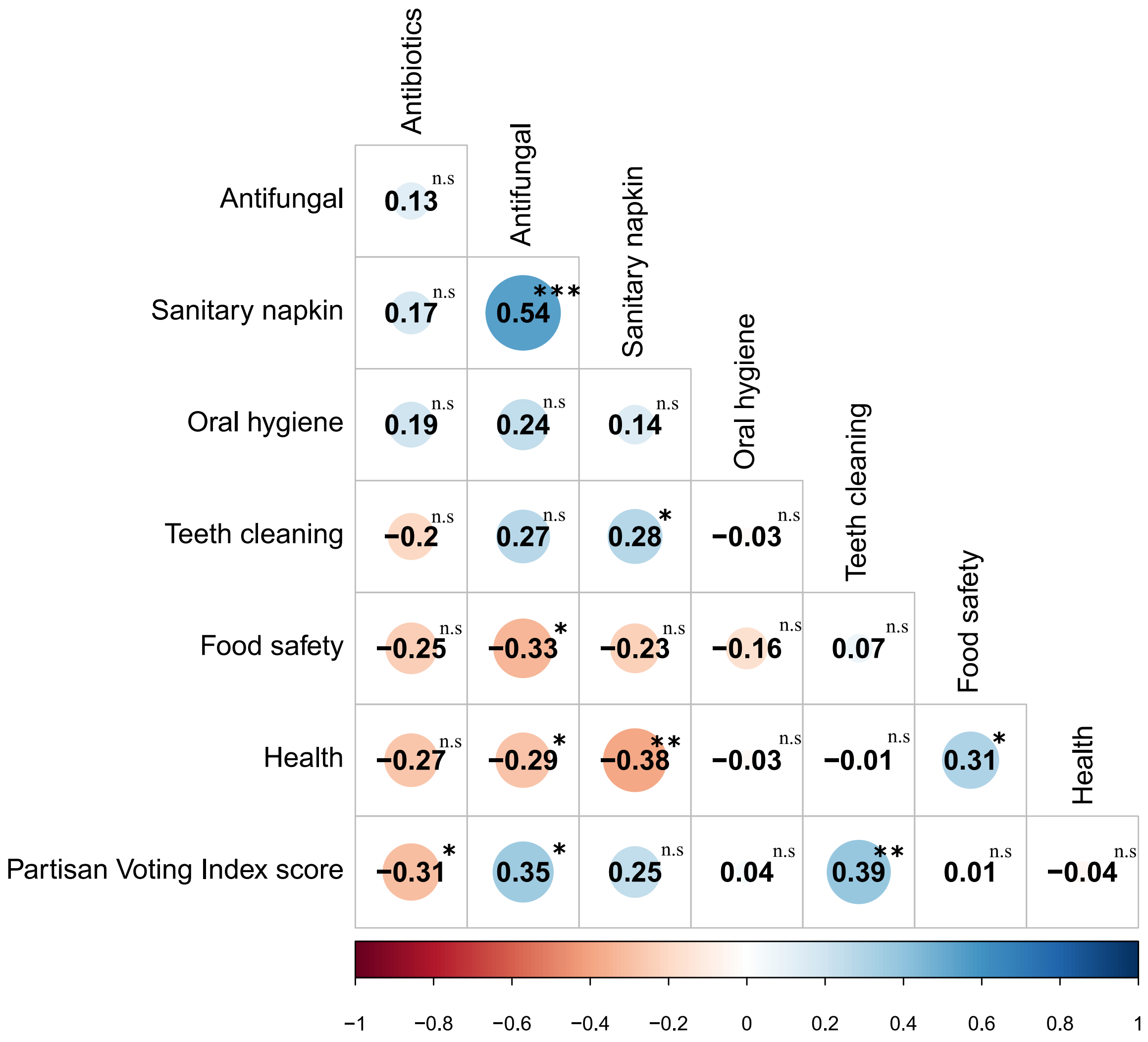

Figure S3. *Non-COVID-related hygiene/sanitary measures analysis: Spearman's rank correlation matrix comparing Relative Search Volume (RSV) of the following non-COVID-related control searches to the 2017 Cook Partisan Voting Index (PVI) for the period 1 January 2020 to 12 December 2020: Antifungal (Drug class), Antibiotics (Drug type), Oral hygiene (Topic), Teeth cleaning (Topic), Food safety (Topic), Health (Topic). Correlation coefficients (ρ) represented graphically and numerically. * p < 0.05, ** p < 0.01, *** p < 0.001*

References

1. Advice for the public on COVID-19 – World Health Organization. Accessed December 20, 2020. https://www.who.int/emergencies/diseases/novel-coronavirus-2019/advice-for-public

2. CDC. Coronavirus Disease 2019 (COVID-19) – Prevention & Treatment. Centers for Disease Control and Prevention. Published November 27, 2020. Accessed December 19, 2020. https://www.cdc.gov/coronavirus/2019-ncov/prevent-getting-sick/prevention.html

3. Johnson DC, Nobles AL, Caputi TL, et al. Monitoring HIV testing and pre-exposure prophylaxis information seeking by combining digital and traditional data. *BMC Infectious Diseases*. 2021;21(1):215. doi:10.1186/s12879-021-05907-0

4. Explore results by region - Trends Help. Accessed January 26, 2021. https://support.google.com/trends/answer/4355212?hl=en

5. WHO | Novel Coronavirus – China. WHO. Accessed December 20, 2020. http://www.who.int/csr/don/12-january-2020-novel-coronavirus-china/en/

6. Holshue ML, DeBolt C, Lindquist S, et al. First Case of 2019 Novel Coronavirus in the United States. *New England Journal of Medicine*. 2020;382(10):929-936. doi:10.1056/NEJMoa2001191
